# What can we learn from patient claims? - A retrospective analysis of incidence and patterns of adverse events after orthopaedic procedures in Sweden

**DOI:** 10.1186/1754-9493-6-2

**Published:** 2012-01-20

**Authors:** Annica Öhrn, Johan Elfström, Hans Tropp, Hans Rutberg

**Affiliations:** 1 Department of Medical and Health Science, Linköping University, Sweden; 2 Patient Safety Unit, County Council of Östergötland, Sweden; 3 Department of Spine Surgery, Faculty of Health Sciences, Linköping University, Sweden

**Keywords:** Insurance claim review, Medical errors, Orthopaedics, Patient safety, Patient admission, Safety management

## Abstract

**Background:**

Objective data on the incidence and pattern of adverse events after orthopaedic surgical procedures remain scarce, secondary to the reluctance for encompassing reporting of surgical complications. The aim of this study was to analyze the nature of adverse events after orthopaedic surgery reported to a national database for patient claims in Sweden.

**Methods:**

In this retrospective review data from two Swedish national databases during a 4-year period were analyzed. We used the "County Councils' Mutual Insurance Company", a national no-fault insurance system for patient claims, and the "National Patient Register at the National Board of Health and Welfare".

**Results:**

A total of 6,029 patient claims filed after orthopaedic surgery were assessed during the study period. Of those, 3,336 (55%) were determined to be adverse events, which received financial compensation. Hospital-acquired infections and sepsis were the most common causes of adverse events (n = 741; 22%). The surgical procedure that caused the highest rate of adverse events was "decompression of spinal cord and nerve roots" (code ABC**), with 168 adverse events of 17,507 hospitals discharges (1%). One in five (36 of 168; 21.4%) injured patient was seriously disabled or died.

**Conclusions:**

We conclude that patients undergoing spinal surgery run the highest risk of being severely injured and that these patients also experienced a high degree of serious disability. The most common adverse event was related to hospital acquired infections. Claims data obtained in a no-fault system have a high potential for identifying adverse events and learning from them.

## Background

Adverse events in connection with medical care are common [1-4] and, based on medical record reviews, several studies have shown that surgical specialities contribute a large share of adverse events [2,3,5]. A Swedish study estimated the number of preventable adverse events as high as 8.6%. Six percent of these adverse events were serious, contributed to death or permanent disability. The surgical disciplines accounted for approximately 62% of the adverse events [6].

Orthopaedic adverse events have been studied in several investigations in recent years [7-11]. Screening for adverse events in medical and nursing records of 395 orthopaedic patients revealed that 16% of the patients experienced an adverse event [10]. Another study found a frequency of perioperative complications and adverse events of 15.7% in connection with lumbar surgery [11]; a further study showed that 21% of patients undergoing total knee arthroplasty experienced adverse events [9].

In addition to medical record reviews, information about adverse events can be obtained by analysing complaints from patients [12-20]. A study from the national Norwegian Patient Compensation System showed that 47% of all patient claims were filed after orthopaedic treatment [12]. In Sweden, patients may claim economic compensation from a national no-fault insurance system, County Councils' Mutual Insurance Company, if they believe they have been injured by the health care system. Approximately 9000 patients file claims every year in Sweden (~9 million inhabitants) and about half of these patients receive economic compensation. Compensation is provided only for injuries that are considered preventable by the medical experts at the insurance company. Orthopaedic surgery is one of the surgical specialities with most claims [13].

The aim of this study was to analyze the nature of adverse events after orthopaedic surgery reported to one national database for patient claims in Sweden.

## Methods

This is a national study conducted in Sweden. Data were obtained from two national registries: the County Councils' Mutual Insurance Company and the National Patient Register. The study period was from 1998 to 2001.

### County Councils' Mutual Insurance Company

The County Councils' Mutual Insurance Company is a national system no-fault insurance system under the Patient Injury Act and contains all patient claims reported from patients or relatives. The system has several characteristics of successful reporting; it is non-punitive, confidential and independent from sanctioning authorities; it uses expert analysis and it provides feedback of claims data to hospitals. The system is unique to the Nordic countries (Norway, Denmark, Finland and Sweden) [13]. Information in the system is not shared with regulatory agencies or professional sanctioning bodies. The patient must report within 3 years from the injury becoming objectively noticeable. The insurance company receives about 9000 claims every year, half of which are accepted for compensation. Injuries associated with orthopaedic treatment resulted in the highest proportion of patient claims sustained (28%), followed by general surgery (20%), obstetrics and gynaecology (11%), anaesthesiology (6%) and internal medicine (5%).

On receiving a claim, the insurance company investigates the case and decides, with the help of medical experts, if compensation should be paid. The compensation from the Insurance Company covers additional treatment costs and income loss caused by the injury. The criteria for compensation are based on the assumption that if an experienced specialist had treated the patient the injury could have been preventable. Compensation is not given for well-known and common complications. The economic compensation from the insurance company is calculated in accordance with the general principle concerning the law of torts in Sweden but the compensation is lower than in the United States. The lower compensation is based on the social system in Sweden, which includes many other types of insurance (for health, traffic, pharmaceutical, work-related etc.).

The database contains information on discharge from hospital, medical specialty, diagnostic codes, surgical procedure codes, patient age and sex. The database also contains information about whether the claim was denied or approved. If approved, the injury type and degree of disability/consequences are recorded. Injury type and consequences are classified by the insurance company. The classification is based on injury criteria defined by the Swedish Patient Injury Act.

During the study period, over 27,000 claims were reported. Of these, 6029 patient claims were attributed to orthopaedic surgery.

### National Patient Register

The National Board of Health and Welfare is a government agency in Sweden under the Ministry of Health and Social Affairs. The National Board of Health and Welfare develop regulations, exercise supervision and maintain health data registers and official statistics. The National Patient Register, which captures 100% of all discharges from Swedish hospitals, includes the number of admissions, the number of admissions per 100,000 inhabitants, number patients treated, number of patients treated per 100,000 inhabitants, length of hospital stay, length of hospital stay per 100,000 inhabitants and average length of hospital stay. From this database, all discharges after orthopaedic treatment during the study period were captured (*n *= 391,579).

Both registries include a field with patients' unique personal identification number and this allows for comparison between the two registries. The number of patient claims in every age category and surgical procedure/diagnosis (three-character ICD 10 codes) were compared with the discharge data from the National Patient Register.

The Regional Research Ethics Committee in Linköping, Sweden, has approved this study. Registration number 03-247.

## Results

During the study period, 6029 patient claims were filed after orthopaedic care. Of those, 3336 (55%) were assessed as an adverse event and were economically compensated accordingly; 2668 patients did not receive economic compensation. The most common reason for rejecting a patient claim was that the injury was assessed as an "unavoidable consequence" of the treatment (27%) or "injury not related to treatment" (24%).

The gender distribution was 57% women and 43% men. The average age of the patients was 51 years (median 53 years, range 0-98 years). Of the patients whose claims were denied 58% were women and 42% were men.

The numbers of claims and compensated claims were highest in the group aged between 50 and 59 years. In proportion to hospital admissions, adverse events were most commonly seen in the group aged 30-39 years. Thereafter, the number of compensated claims decreased despite an increase in the number of hospital discharges. An adverse event resulting in economic compensation occurred in 0.9% of all discharged orthopaedic patients (Table 1).

**Table 1:** Age distribution. Number of orthopaedic patient claims, adverse events and hospital discharges (inpatient and the 20 most frequent orthopaedic diagnostic classifications) in Sweden during the period 1998-2001

Age (years)	Number of patient claims	Number of adverse events (compensated claims)	Number of discharges	Patient claim rate (%)	Adverse events rate (%)
0-9	99	64	11,373	0.9	0.6
10-19	306	172	18,306	1.7	0.9
20-29	553	282	16,967	3.3	1.7
30-39	802	426	20,417	3.9	2.1
40-49	885	479	24,569	3.6	2
50-59	1243	692	42,663	2.9	1.6
60-69	908	533	54,698	1.7	1
70-79	848	500	93,684	0.9	0.5
> 80	385	213	108,902	0.4	0.2
**Total**	**6029**	**3361**	**391,579**	**1.5**	**0.9**

The most common location of injuries was bones and joints (1126 of 3336; 50%), followed by injuries to nerves (775 of 3336; 23%). The most common types of adverse events according to the classification in the Insurance Company's register were hospital-acquired infections and sepsis 741 of 3336; 22%) followed by delay in diagnosis and/or treatment (577 of 3336; 17%) (Tables 2 and 3).

**Table 2:** Type of adverse events in orthopaedics in Sweden during the period 1998-2001.

Type of adverse events	Number of adverse events (%)	Proportion of expense (%)
Hospital-acquired infections and sepsis	741 (22)	25.5
Delayed diagnosis and/or treatment	577 (17)	23.5
Others injuries	358 (10)	8.5
Fracture/dislocation	291 (9)	5.5
Severance (e.g. nerve)	271 (8)	13.2
Pain	181 (5)	2.7
Compression injury (e.g. cast, table)	178 (5)	2.5
Bleeding	40 (1)	2.1
Thrombosis, embolism	36 (1)	0.3
Unspecified other local injury	688 (20)	16.2
**Total**	**3361**	**100**

**Table 3:** The surgical procedures most frequently leading to adverse events and the number for each type of adverse events

Type of adverse events	Primary prosthetic replacement of hip joint (code NFB**)	Decompression of spinal cord and nerve roots (code ABC**)	Primary prosthetic replacement of knee joint (code NGB**)	Fracture surgery of femur (code NFJ**)	Fracture surgery of ankle and foot femur (code NHJ**)	Fracture surgery of knee and lower leg (code NGJ**)
Hospital-acquired infections and sepsis	72	38	73	31	32	18
Delayed diagnosis and/or treatment	19	18	9	9	13	14
Others injuries	34	27	17	7	6	16
Fracture/dislocation	42	1	13	23	7	2
Severance (e.g. nerve)	39	16	16	1	6	9
Pain	15	9	3	3	5	3
Compression injury (e.g. cast, table)	20	10	14	10	8	4
Bleeding	2	13	2	0	0	0
Thrombosis, embolism	2	2	3	0	2	0
Unspecified other local injury	59	34	37	21	24	26
**Total**	**304**	**168**	**187**	**105**	**103**	**92**

The surgical procedure that caused the highest rate of adverse events was "decompression of spinal cord and nerve roots" (code ABC**), with 168 adverse events of 17,507 hospitals discharges (1%). These adverse events also resulted in a higher degree of disability. One in five (36 of 168; 21.4%) injured patient was seriously disabled or died (Table 4). The highest number of adverse events was observed after primary prosthetic replacement of hip and knee joints, which are common surgical procedures (Table 4).

**Table 4:** The surgical procedures most frequently leading to adverse events and the number of hospital discharges and degree of disability in Sweden during the period 1998-2001

Surgical procedure classification (three-character ICD 10 code)	Number of hospital discharges	Number of adverse events (%)	Degree of disability (no. of patients)				
			
			Sick leave ≤ 3 months	1-15%	16-30%	> 30%	Death
Primary prosthetic replacement of hip joint (NFB**)	50,733	304 (0.6)	18	259	22	4	1
Primary prosthetic replacement of knee joint (NGB**)	23,600	187 (0.8)	12	163	9	2	1
Decompression of spinal cord and nerve roots (ABC**)	17,507	168 (1.0)	12	120	12	22	2
Fracture surgery of femur (NFJ**)	68,123	105 (0.2)	9	80	14	1	1
Fracture surgery of ankle and foot femur (NHJ**)	22,490	103 (0.5)	8	92	2	2	0
Fracture surgery of knee and lower leg (NGJ**)	12,594	92 (0.7)	5	84	3	0	0

## Discussion

This retrospective study describes adverse events after orthopaedic surgery. The following findings were noted: the high number of adverse events caused by infections after orthopaedic surgery and the high degree of serious disability after adverse events in connection with spine surgery. Another important finding was the relatively high number of adverse events causing permanent disability in the two large groups involving primary knee and hip prosthetic replacement. The finding that hip and knee replacement and intervertebral disc surgery were the most common procedures resulting in sustained claims is in accordance with what has been reported in a small American study [21].

The nature of the national systems for compensation of adverse events may influence the extent to which review of claims data can be used to learn more about adverse events. Claims data in the United States are primarily used by litigation managers, attorneys, and others for determining legal liability. Previous studies have shown little relationship between errors and malpractice claims [22]. The no-fault system in Sweden, in contrast, does not place the responsibility for a medical error on an individual practitioner and may reduce barriers to filing for compensation and increase the probability that an error is disclosed [13]. The relative rate of claims in Sweden exceeds that of countries with a litigation system, suggesting that its reporting system may contribute to making claims data more useful for identifying and learning more about adverse events.

In Sweden, a third of all patient claims involve orthopaedic treatment, whereas in the United States the percentage of orthopaedic claims is around 14% of the total claims [20]. Our finding that orthopaedic surgery had the highest share of claims among all specialities at the insurance company is in accordance with what has been reported before in a study from another Nordic country with a no-fault system [12].

Jena et al. [20] reported malpractice claims according to speciality in the United States and found that neurosurgery, thoracic-cardiovascular surgery, general surgery and orthopaedic surgery were the specialities with the highest probability of claims. In Sweden, the following specialities have the highest probability of a claim: hand surgery, orthopaedics, cardiothoracic surgery and neurosurgery [13].

The high rate claims in connection with orthopaedic surgery might be due to high patient expectations or because complications are more obvious than in other specialities. Knee and hip prosthesis surgery are considered by the public to be very safe routine procedures and if the result is not what the patient expected, it may result in a claim. Wong et al. [7] reported that the reasons for adverse events in orthopaedics are multifaceted; for example, communications failure, equipment and/or instrumentation problems in the operating room, improper technique and/or physician impairment. However, these reasons are no different for other surgical specialties.

The number of patient claims and adverse events in relation to hospital admissions varied tenfold between different age groups in this study. In the most elderly group (> 80 years), fewer adverse events were found despite the fact that this age group is the largest in most orthopaedic departments. This could be explained in two ways: either elderly patients undergo less complex procedures with lower risk or they are less prone to claim economic compensation. The high rate of claims in the group aged 20-59 years can probably be explained by a higher tendency to report injury, most likely related to loss of income during working years.

This study reveals that almost 1% of all orthopaedic patients receive compensation for adverse events assessed as preventable. Even though the number of patient claims is increasing slightly year by year, the actual number of adverse events is probably much higher, as indicated by the results of a national study showing that 8.6% of patients experience preventable adverse events [6].

The frequency of hospital-acquired infections and sepsis after orthopaedic surgery in this study is in agreement with findings in an Australian study on adverse event in general surgery and orthopaedic surgery [5]. A high rate of hospital-acquired infections and sepsis is also reported from the United Kingdom in studies on adverse events [3,4] but there is a paucity of studies on the frequency of infections as a cause of adverse events in orthopaedics. In our study, 22% of the patients were awarded compensation on the basis of infection, which is higher than previously found in a Norwegian study (8.1%) [12]. At least in Sweden, retrospective analysis of patient claims data seems to be able to identify and provide additional information on the consequences of hospital-acquired infections. When we investigated 113 cases of injury leading to serious disability and compared these injuries with a mandatory hospital-based sentinel event reporting database, we found that none of the 19 claims involving health care-associated infections that led to permanent disability were reported by the providers [23]. Furthermore, when reviewing patient claims after spine surgery, health care-associated infections were the second most common adverse events leading to compensation [24].

Procedures to decompress spinal nerve roots and the spinal cord result in adverse events more often than other surgical procedures [15]. In our study, nearly one-fourth of the patients suffered serious disability after spine surgery. In a study with the aim to describe and analyse the outcome after spine surgery, claims data were compared from the County Councils' Mutual Insurance Company with data from a national register and medical records. It was found that dural lesions were a common, but underreported, complication and an important reason for problems contributing to high levels of disability [24].

Our study is a retrospective analysis of the contents of the County Councils' Mutual Insurance Company database. The variables used by the insurance company are primarily designed for the reimbursement procedure and not for medical analysis. The data set is complete only for patients who received economic compensation. Despite these shortcomings, we consider the database to be an important source of information on adverse events. The study is a survey of a whole range of orthopaedic injuries but has nevertheless identified some risk groups. In order to identify underlying causes of adverse events in connection with hip and knee replacement, it would be interesting to compare claims data with data from national registers and medical records.

We conclude that patients undergoing spinal surgery were subjected to high risk of being severely injured and that these patients also experienced a high degree of serious disability. The most common adverse event was related to hospital acquired infections. Claims data obtained in a no-fault system have a high potential for identifying adverse events and learning from them.

## Competing interests

The authors declare that they have no competing interests.

## Authors' contributions

AÖ, JE, HT and HR defined the research goal and designed the methods. AÖ collected the data. AÖ, HT and HR analyzed and interpreted the result. All the authors contributed to writing, read and approved the final manuscript.
